# Disturbed chromosome segregation and multipolar spindle formation in a patient with *CHAMP1* mutation

**DOI:** 10.1002/mgg3.303

**Published:** 2017-07-12

**Authors:** Nobuhiko Okamoto, Yuki Tsuchiya, Ichiro Kuki, Toshiyuki Yamamoto, Hirotomo Saitsu, Daiju Kitagawa, Naomichi Matsumoto

**Affiliations:** ^1^ Department of Medical Genetics Osaka Women's and Children's Hospital Osaka Japan; ^2^ Division of Centrosome Biology Department of Molecular Genetics National Institute of Genetics Mishima Japan; ^3^ Department of Genetics School of Life Science The Graduate University for Advanced Studies (SOKENDAI) Mishima Japan; ^4^ Department of Pediatric Neurology Osaka City General Hospital Osaka Japan; ^5^ Tokyo Women's Medical University Institute for Integrated Medical Sciences Tokyo Japan; ^6^ Department of Biochemistry Hamamatsu University School of Medicine Hamamatsu Japan; ^7^ Department of Human Genetics Yokohama City University Graduate School of Medicine Yokohama Japan

**Keywords:** *CHAMP1*, intellectual disability (ID), lymphoblastoid cell, multipolar spindle

## Abstract

**Background:**

Patients with intellectual disability (ID) typically exhibit significant defects in both intelligence and adaptive behavior. Aberration of several genes involved in proper progression of mitosis has been reported to underlie ID. Here, we report a new patient with a novel mutation of *CHAMP1*.

**Methods:**

Whole exome sequencing (WES) analysis was performed. We isolated lymphoblast cells from the *CHAMP1* patient and observed chromosome segregation.

**Results:**

We identified a de novo frameshift mutation in *CHAMP1*. We find that these cells exhibit an increase in centrosome number and resulting multipolar spindle formation. The phenotypes observed in the patient's lymphoblastoid cells were presumably because of cytokinesis failure. We also confirm the identical phenotypes in human culture cells depleted of CHAMP1.

**Conclusion:**

*CHAMP1* encodes a protein regulating kinetochore–microtubule attachment and chromosome segregation. These data strongly support that *CHAMP1* mutations cause ID, and suggest that CHAMP1 is critical for progression of cytokinesis and maintenance of centrosome number.

Intellectual disability (ID) is a neurodevelopmental disorder characterized by substantial defects in both intelligence and adaptive behavior. Aberration in mitotic process of neural progenitor cells has been considered as one of causes for ID, because mutations in several genes associated with mitotic process have been found in ID.

In most animal cells, the centrosome consisting of two cylindrical centrioles surrounded by pericentriolar material (PCM) functions as the microtubule‐organizing centers (MTOC). Tight control of the centrosome number in a cell is essential for robust formation of bipolar spindles and proper chromosome segregation during mitosis (Gönczy 2015). Indeed, centrosome amplification can lead to severe problems, such as multipolar spindle formation, chromosome missegregation, and genomic instability, which are generally known as the hallmarks of cancer. Abnormal centrosome and spindle morphology are also found in a patient with autosomal recessive primary microcephaly (MCPH), which typically causes intellectual disability (Nigg and Raff 2009). The mutations of centrosome‐related genes identified in MCPH are likely to be loss of function. However, it has been suggested that centrosome amplification could also cause MCPH phenotypes (Arquint and Nigg 2014), suggesting that the strict regulation on the centrosome copy number is critical for proper brain development.

Formation of multipolar spindles could be induced by various defects in mitotic spindle pole integrity. For example, human cells with centriole overduplication or cytokinesis failure undergo multipolar spindle formation that is associated with centrosome amplification during mitosis. In other cases, precocious centriole disengagement or PCM fragmentation before or during mitosis could result in multipolar spindle formation (Maiato and Logarinho 2014).

Chromosome alignment maintaining phosphoprotein 1 (CHAMP1) was initially reported to be a protein regulating kinetochore–microtubule attachment (Itoh et al. 2011). Interestingly, they suggested that the C‐terminal region of CHAMP1 containing the zinc‐finger domains was important in regulating CHAMP1 localization to chromosomes and the mitotic spindle. Moreover, a large‐scale sequencing study in 1133 individuals with ID revealed that de novo *CHAMP1* alterations are involved in developmental disorders (Wright et al. 2015). The study included two patients with autosomal dominant mental retardation 40 (MRD40) (OMIM#616579), who had mutations in the *CHAMP1* gene. Another study identified *de novo* deleterious mutations in *CHAMP1* in five unrelated individuals affected by ID (Hempel et al. 2015). Note that all mutations were predicted to lead to the loss of the zinc finger domains regulating CHAMP1 localization to chromosomes and the mitotic spindle. Furthermore, two independent studies reported further 11 patients with *de novo* mutations in *CHAMP1* (Isidor et al. 2016) (Tanaka et al. 2016).

In this study, we report a novel patient with *CHAMP1* mutation. Consistent with the previous reports, we observed chromosome segregation errors during mitosis of the patient lymphocyte cells. Moreover, we found the cytokinesis failure and accompanying centrosome amplification phenotypes in the patient's cells. These data illustrate novel aspects of the phenotypes caused by *CHAMP1* mutations.

The patient, a 6‐year‐old boy, was the first child of Japanese parents with no consanguinity. He was delivered at full term with a birth weight of 2400 g (−2.3 SD), body length 47 cm (−1.6 SD), and OFC 32 cm (−1.3 SD). He had feeding difficulty and failure to thrive. Delayed motor development was apparent from the infantile period, and he could not walk until the age of 4 years. Severely delayed acquisition of language skills and intellectual disability were noted. He showed spasticity of lower extremities.

At 4 years, the patient had visited the pediatric neurology department because of frequent complex partial seizures. EEG showed spike activities. Seizures were controlled by antiepileptic drugs. Routine laboratory investigations and metabolic survey gave normal results. Standard karyotyping using peripheral blood showed no abnormalities. Brain magnetic resonance imaging showed cavum septum pellucidum, cavum vergae, cerebral atrophy, and decreased white matter volume (Fig. 1A).

**Figure 1:**
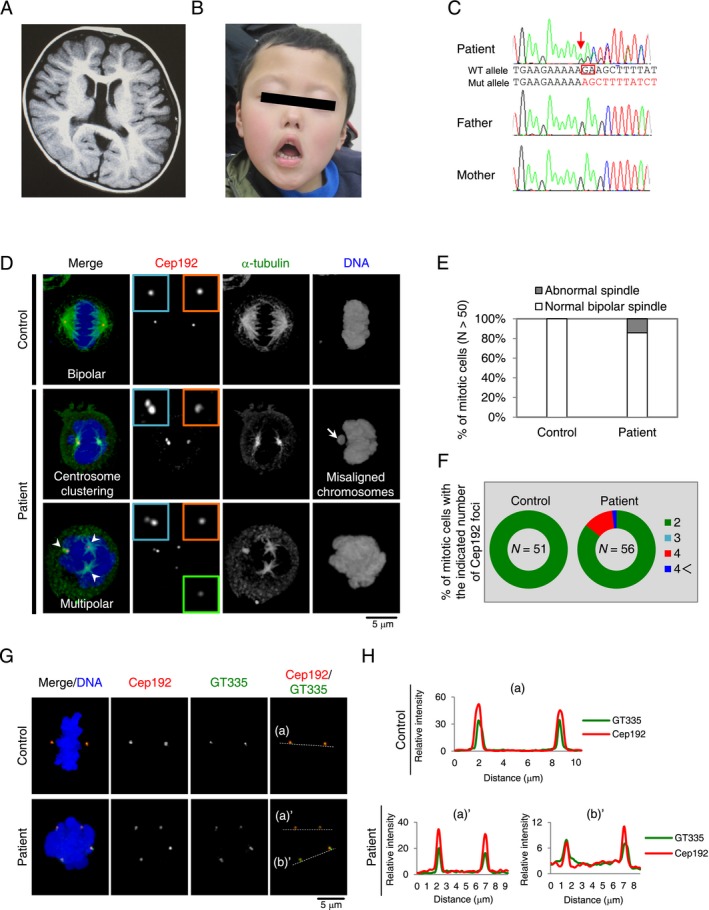
Clinical features and cytological abnormalities in a patient with *CHAMP1* mutation. (A) Brain MRI showed cavum septum pellucidum, cavum vergae, cerebral atrophy, and decreased white matter volume. (B) Patient at 6 years old. Craniofacial dysmorphic features, including microcephaly, brachycephaly, sparse eyebrow, prominent eyes, down slanting palpebral fissures, ectropion of lower eyelids, flat nasal bridge, anteverted nostrils, short philtrum, down turned corners of mouth were noted. (C) Abnormal spindle formation and chromosome misalignment of patient cells with *CHAMP1* mutation. Lymphoblast cells from a healthy person and a patient with *CHAMP1* mutation were stained with antibodies against α‐tubulin (green) and Cep192 (red). Nuclei are shown in blue. Insets show approximately twofold‐magnified views of Cep192 signals. White arrow indicates misaligned chromosomes. White arrowheads indicate excess Cep192 foci representing multipolar spindle. (D) Histograms represent frequency of mitotic cells with normal bipolar and abnormal spindles in lymphoblast cells from a healthy person and a patient with *CHAMP1* mutation. (E) Pie graphs represent frequency of mitotic cells with the indicated Cep192 foci in each condition. (F) Centrosome amplification of patient cells with *CHAMP1* mutation. Lymphoblast cells from a healthy person and a patient with *CHAMP1* mutation were stained with antibodies against GT335 (green) and Cep192 (red). Nuclei are shown in blue. (G) The graph shows the signal intensity of GT335 (green) and Cep192 (red) at the centrosomes along the dotted line in representative panels. All Cep192 signals were overlapped with GT335 signals (*n *=* *10 cells).

At the age of 6 years and 3 months, the patient was evaluated for further genetic studies. On physical examination, body weight of 17.5 kg (‐1.6 SD), height of 105 cm (‐2 SD), head circumference of 49 cm (‐1.5 SD) were observed. Craniofacial dysmorphic features, including brachycephaly, sparse eyebrow, prominent eyes, down slanting palpebral fissures, ectropion of lower eyelids, flat nasal bridge, anteverted nostrils, a short philtrum, down turned corners of mouth were noted (Fig. 1B). He also showed small penis and small hands and feet. Ophthalmological investigations revealed astigmatism and amblyopia. He presented abnormal behavior. He was hyperactive and nodded repetitively for throughout the day. This movement was not accommodated with epileptic EEG discharge. He still has complex partial seizures every day. Array‐CGH revealed no significant copy number changes.

Although it has recently been reported that CHAMP1 somehow regulates kinetochore–microtubule attachment (Itoh et al. 2011), and also that mutations in *CHAMP1* cause ID (The Deciphering Developmental Disorders Study 2015; (Hempel et al. 2015) (Isidor et al. 2016) (Tanaka et al. 2016), the biological evidence for linking between the patient mutations in *CHAMP1* and ID is insufficient.

WES analysis was performed as described previously (Saitsu et al. 2016). Trio‐WES identified a *de novo* frameshift mutation of *CHAMP1* (NM_032436.2: c.2068_2069delGA, p.Glu690Serfs*5) (Fig. 1C). Interestingly, this mutation may lead to an early truncating protein lacking the zinc finger domains that were important for CHAMP1 localization to chromosomes and the mitotic spindle. Note that this variant was never observed in ExAC, EVS, 1000 Genome, HGVD, and our in‐house exome database (*n* = 575).

To identify the cytological abnormalities in patient cells with *CHAMP1* mutation, we isolated and analyzed lymphoblast cells from the blood cells of a healthy person and a patient with *CHAMP1* mutation. To investigate overall mitotic features in these cells, we used immunofluorescence analyses with specific antibodies against endogenous centrosomal protein of 192 kDa (Cep192) and α‐tubulin to mark centrosomes and microtubules, respectively. While control mitotic cells had two Cep192 foci at the opposite sides and formed functional bipolar spindles, we found that patient's cells with the *CHAMP1* mutation showed formation of pseudo‐bipolar spindles with centrosome clustering or multipolar spindles during mitosis (14% of the *CHAMP1* patient cells with abnormal spindle formation, compared with 0% of control cells; Fig. 1D,E). Consistent with the previous studies, we also observed that 3.6% of the patient cells showed misaligned chromosomes during mitosis (Fig. 1D). Importantly, we found that most multipolar spindles in the *CHAMP1* patient cells harbored four foci of Cep192, all of which seemed to have MTOC activity (Fig. 1F).

Cep192 is known as a component of the centriole as well as pericentriolar material (PCM) which organize a functional centrosome (Sonnen et al. 2013). Therefore, to distinguish whether these excess Cep192 dots reflect centrosome amplification, centriole disengagement, or PCM fragmentation, we next performed immunofluorescence analyses using specific antibodies against centriolar glutamylated microtubules (GT335) and Cep152, a mother centriole marker, in lymphocyte cells (Tsuchiya et al. 2016). We found that, in the *CHAMP1*‐mutant patient's cells, all Cep192 foci on the multipolar spindle poles contained GT335 dots (Fig. 1G,H). Using triple staining analysis, we further confirmed that these excess foci marked with Cep152 colocalized with a pair of adjacent GT335 dots and served as a MTOC (Fig. S1). These data indicate that multipolar spindle formation in our patient's cells is because of the increased number of centrosomes during mitosis.

Although it has been reported that depletion of CHAMP1 results in formation of multipolar spindles in human cells (Itoh et al. 2011), its underlying mechanism remains unclear. To address this, we next investigated the phenotype provoked by RNAi‐mediated depletion of CHAMP1 in human cancer cell line. Using siRNAs targeting different sequences of *CHAMP1* ORF (open reading frame), we confirmed that the depletion of CHAMP1 induced chromosome misalignment in metaphase, which was further confirmed in different human cell lines, HeLa and U2OS cells (Fig. 2A–C). These results are consistent with the previous observation that CHAMP1 is important for proper chromosome alignment during mitosis (Itoh et al. 2011).

**Figure 2:**
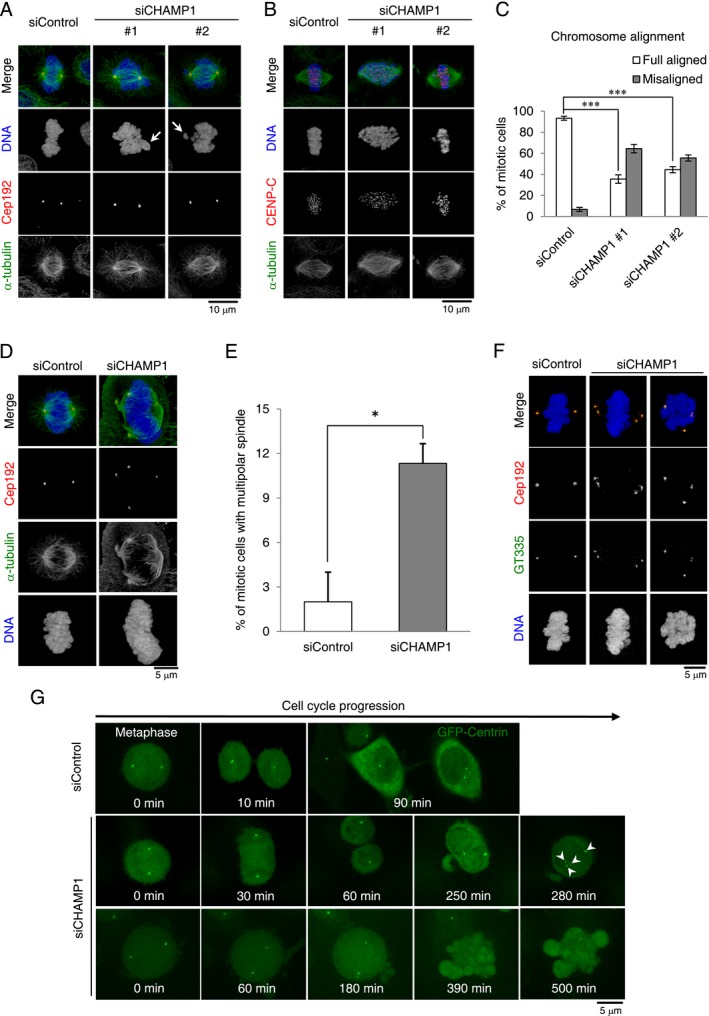
Depletion of CHAMP1 causes chromosome misalignment and the increased number of centrosomes during mitosis in human cancer cells. Depletion of CHAMP1 induces chromosome misalignment and instability of kinetochore–microtubules. U2OS (A) and HeLa (B) cells treated with control siRNA or siRNA targeting endogenous CHAMP1 for 72 h were stained with the indicated antibodies. Nuclei are shown in blue. White arrow indicates misaligned chromosomes. (C) Histograms represent frequency of mitotic HeLa cells with the indicated category in each condition. Values are mean percentages ± SEM from three independent experiments (*n *=* *30 for each condition). ***P < 0.001 (two‐tailed *t*‐test). (D) Depletion of CHAMP1 promotes formation of the excess centrosome foci during mitosis. U2OS cells treated with control siRNA or siRNA targeting endogenous CHAMP1 for 48 h were stained with the indicated antibodies. Nuclei are shown in blue. Cells were treated with MG132 for the last 2 h. (E) Histograms represent frequency of mitotic cells with multipolar spindles in each condition. Values are the mean percentages ± SEM from three independent experiments (*n *=* *50 for each condition). *P < 0.05 (two‐tailed *t*‐test). (F) Centrosome amplification in CHAMP1‐depleted cells. HeLa cells treated with control siRNA or siRNA targeting endogenous CHAMP1 for 48 h were stained with the indicated antibodies. Nuclei are shown in blue. Cells were treated with MG132 for the last 2 h. (G) Depletion of CHAMP1 causes cytokinesis failure. Live imaging of cycling HeLa cells expressing GFP‐centrin1 (green) and treated with control siRNA or CHAMP1 siRNA. Scale bar = 5 μm. White arrowheads indicate the increased number of centrosomes. 5.8% of control cells showed cell death (*n *=* *17), compared with 43.3% of CHAMP1‐depleted cells; 10% of CHAMP1‐depleted cells showed cytokinesis defects (*n *=* *30).

We next aimed to clarify whether the formation of multipolar spindles during mitosis is due to the existence of excess centrosomes. First, we confirmed, in HeLa cells, that depletion of CHAMP1 induced formation of multipolar spindles with excess Cep192 foci as was observed in the *CHAMP1*‐mutant patient lymphocyte cells (11% of CHAMP1‐depleted cells with multipolar spindle formation, compared with 2% of control cells; Fig. 2D,E). We also noticed the clear trend that most *CHAMP1*‐depleted cells harbored four Cep192 foci that appeared to act as MTOCs during mitosis. In addition, these Cep192 foci colocalized with GT335 signals, suggesting that all multipolar spindle poles contain a pair of centrioles (Fig. 2F). As expected from the observation of the *CHAMP1* patient phenotypes, we next sought to obtain direct evidence that centrosome amplification upon CHAMP1 depletion is a secondary effect of cytokinesis failure. Using live cell imaging with HeLa cells expressing GFP‐centrin that marks centrioles, we found that depletion of CHAMP1 indeed caused cytokinesis failure (~10% of the cells treated with CHAMP1 siRNA; Fig. 2G). In CHAMP1‐depleted cells, we also observed frequent mitotic catastrophe probably due to defects in kinetochore–microtubule attachment (43.3% of CHAMP1‐depleted cells, compared with 5.8% of control cells; Fig. 2G). Taken together, these data strongly suggest that multipolar spindle formation seen in CHAMP1‐depleted cells is due to centrosome amplification following cytokinesis failure.

Hempel et al. (2015) and Isidor et al. (2016) reported individuals with a *de novo* deleterious *CHAMP1* mutation. The individuals showed ID and delayed motor development with a particularly severe delay in speech development. Muscular hypotonia, feeding difficulties and orofacial hypotonia were often observed. Our patient showed similar clinical manifestations. Dysmorphic features, including round face, facial hypotonia, hypertelorism, upslanting palpebral fissures, low‐set ears, short philtrum, high arched palate, tented upper lip, everted lower lip, and pointed chin were often observed. A friendly behavior was described in many individuals. Some individuals displayed stereotypic movements. Our patient showed similar dysmorphic features and behavior abnormalities.

Brain MRI abnormalities were described in some patients reported by Hempel et al. (2015) and Isidor et al. (2016). Mild brain atrophy with cerebellar cortical dysplasia, slightly delayed myelination, thickening of the corpus callosum, etc., were described. Brain MRI in our patient was characteristic. Cavum septum pellucidum, cavum vergae, cerebral atrophy, and decreased white matter volume were noted.

Our work suggests that depletion of CHAMP1 causes not only misaligned chromosomes, but also centrosome amplification following cytokinesis failure. Symmetrical and asymmetrical divisions of the neural progenitor cells are crucial steps during embryonic neurogenesis. Therefore, it is plausible that such mitotic defects in neural progenitor cells could result in significant reduction of neural progenitor pool and defective neural development. We also note that the cytological analysis used in this study could be useful for diagnosis of an ID patient judging whether a symptom is because of loss‐of‐function mutations in *CHAMP1*.

How could depletion of CHAMP1 cause formation of multipolar spindles? Our work indicates that depletion of *CHAMP1* results in mitotic delay and eventual cell death, or otherwise in cytokinesis failure (Fig. 2G). Given that an ID patient also has heterozygous truncating mutations in the *CHAMP1* gene, we speculate that the expression level of CHAMP1 is a critical factor for proceeding mitosis properly. Further detailed studies will be required to understand why a certain protein expression level of CHAMP1 is needed for the proper progression of the cell divisions during brain development.

## Conflict of Interest

The authors have no conflicts of interest to disclose.

## Ethics

Written informed consent for genetic analysis was obtained from the parents according to the ethical guidance of the institutional review board. The ethics committees of the each institution approved this study.

## Supporting information


**Figure S1** The increased number of centrosomes in a patient with *CHAMP1* mutation.
