# Supplementary material for: Disturbed chromosome segregation and multipolar spindle formation in a patient with CHAMP1 mutation
Source: Mol Genet Genomic Med. 2017 Jul 12;5(5):585–91. doi: 10.1002/mgg3.303 (PMC5606869; doi:10.1002/mgg3.303)
Supplement: Supplementary file 1 — Figure S1 The increased number of centrosomes in a patient with CHAMP1 mutation. [file MGG3-5-585-s001.pdf]

Supporting Information for the article:

Disturbed chromosome segregation and multipolar spindle formation in a patient with *CHAMP1* mutation

Nobuhiko Okamoto[1†*], Yuki Tsuchiya[2,3†], Ichiro Kuki[4], Toshiyuki Yamamoto[5], Hirotomo Saitsu[6], Daiju Kitagawa[2,3*], Naomichi Matsumoto[7*]

1 Department of Medical Genetics, Osaka Women's and Children's Hospital , Osaka, Japan

2 National Institute of Genetics, Department of Molecular Genetics, Division of Centrosome Biology, Mishima, Shizuoka 411-8540, Japan.

3 Department of Genetics, School of Life Science, The Graduate University for Advanced Studies (SOKENDAI), Mishima, Shizuoka 411-8540, Japan.

4 Department of Pediatric Neurology, Osaka City General Hospital, Osaka, Japan.

5 Tokyo Women's Medical University Institute for Integrated Medical Sciences, Tokyo, Japan

6 Department of Biochemistry, Hamamatsu University School of Medicine, Hamamatsu, Japan

7 Department of Human Genetics, Yokohama City University Graduate School of Medicine, Yokohama, Japan

† Equal contribution

* co-corresponding authors

Address correspondence to:

Nobuhiko Okamoto

Department of Medical Genetics, Osaka Women 's and Children' s Hospital

e-mail: okamoto@osaka.email.ne.jp

Daiju Kitagawa

National Institute of Genetics, Department of Molecular Genetics, Division of Centrosome Biology

e-mail: dkitagaw@nig.ac.jp

Naomichi Matsumoto

Department of Human Genetics, Yokohama City University Graduate School of Medicine, Yokohama, Japan

e-mail: naomat@yokohama-cu.ac.jp

SUPPORTING INFORMATION (Table of contents)

SUPPORTING METHODS 4

1. Cell culture 4

2. RNA interference..4

3. Antibodies...4

4. Microscopy...5

5. Live cell imaging..6

SUPPORTING FIGURE 8

Supp Figure S1. The increased number of centrosomes in a patient with *CHAMP1* mutation...8

SUPP. REFERENCES...9

SUPP. METHODS

1. Cell culture

Preparation of Lymphoblast cells. Lymphoblast cells from the blood cells of a healthy person and a patient with *CHAMP1* mutation were cultured in Roswell Park Memorial Institute (RPMI) 1640 medium containing 10% fetal bovine serum (FBS) at 37 ºC in a 5% CO_2 atmosphere. HeLa and U2OS cells were obtained from the ECACC (European collection of cell cultures). HeLa cells stably expressing GFP-centrin1 were generated as previously described[Piel, M et al. 2000]. HeLa and U2OS cells were cultured in Dulbecco's modified Eagle's medium (DMEM) with 10% FBS at 37 ºC in 5% CO_2 incubator.

2. RNA interference

The following siRNAs were used: Silencer Select siRNA (Life Technologies) against ZNF828 (known as CHAMP1) #1 (s49268), ZNF828 #2 (s49269) and negative control #1 (4390843). Transfection of siRNA into HeLa and U2OS cells was conducted using Lipofectamine RNAiMAX (Life Technologies). Unless otherwise noted, silencer Select siRNA (Life Technologies) against ZNF828 (known as CHAMP1) #2 (s49269) was used in this study and the transfected cells were analyzed 48-72 hours after transfection with siRNA.

3. Antibodies

The following primary antibodies were used in this study: Guinea pig polyclonal

antibodies against CENP-C (MBL, PD030, IF 1:1000), Rabbit polyclonal antibodies against Cep192 (Bethyl laboratories, A302-324A, IF 1:1000), Cep152 (Bethyl laboratories, A302-480A, IF 1:1000); mouse monoclonal antibodies against, Polyglutamylated tubulin (GT335, mAb) (AdipoGen, AG-20B-0020-C100, IF 1:5000), α-tubulin (Sigma-Aldrich, DM1A, IF1:1000); Alexa 647- labeled Cep152 (Bethyl laboratories, A302-480A, IF 1:200) was generated with Alexa Fluor labeling kits (Life Technologies) and used for three color staining in Supplementary Figure S1. The following secondary antibodies were used: Alexa Fluor 488 goat anti-mouse IgG (H+L) (Molecular probes, A-11001, 1:500), Alexa Fluor 568 goat anti-rabbit IgG (H+L) (Molecular probes, A-11011, 1:500) for IF.

4. Microscopy

For immunofluorescence analysis, HeLa and U2OS cells cultured on coverslips (Matsunami: No 1 for confocal microscope) were fixed using -20ºC methanol for 10 minutes and washed with PBS. The cells were permeabilized after fixation with PBS/0.05% TritonX-100 (PBSX) for 5 minutes three times, and incubated for blocking in 1% BSA in PBSX for 30 minutes at room temperature (RT). The cells were then incubated with primary antibodies for 24 hours at 4 °C, washed with PBSX three times, and incubated with secondary antibodies for 1 hour at RT. The cells were thereafter washed with PBSX twice, stained with 0.2 µg ml^{-1} Hoechst 33258 (DOJINDO) in PBS for 5 minutes at RT, washed again with PBSX and mounted onto glass slides.

For specimen slide preparation of Lymphoblast cells, cell suspension was mixed

with Smear Gell (GenoStaff) and spread on the surface of slide. The same steps as above were repeated to perform immunofluorescence analysis of specimen slide preparation.

Counting the number of immunofluorescence signals was done using an Axioplan2 fluorescence microscope (Carl Zeiss) with a 100x/1.4 NA plan-APOCHROMAT objective. We assessed cells from several fields for each experiment. The investigators were normally blinded to the sample ID during experiments and outcome assessment. Once a field was determined, we counted all cells which match the criteria within the field.

Confocal microscopy images were taken by the Leica TCS SP8 HSR system equipped with a Leica HCX PL APO ×63 / 1.4 oil CS2 objectives and excitation wavelength 405, 488, 561 and 647 nm. Scan speed was set to 200 Hz in combination with 5 fold line average in 1024 x 500 format. The images were collected at 300 nm z steps. For deconvolution, Huygens essential software (SVI; Scientific Volume Imaging) was used.

5. Live cell imaging.

A Confocal Scanner Box, Cell Voyager CV1000 (Yokogawa Electric Corp) equipped with a 63× oil immersion objective lens and the stage incubator for 35mm dish was used for live cell imaging. HeLa cells stably expressing GFP-centrin1 were treated with control siRNA or CHAMP1 siRNA for 24 hours and cultured on 35 mm glass-bottom dishes (MatTek Co.) at 37 ºC in a 5% CO_2 atmosphere. Images were taken

by Back-illuminated EMCCD camera. After 24 hours from transfection, the cells were visualized every 10 min over 24–36 hours. The images were collected at 1 μm z steps (from 25 to 30 Z-planes and generated using ImageJ (National Institutes of Health)).

SUPPORTING FIGURE

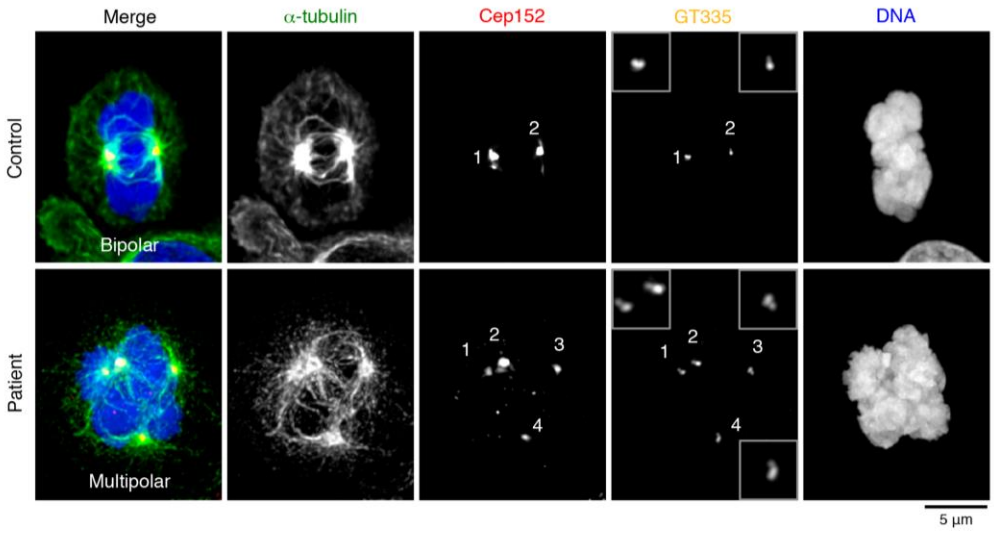

Supp Figure S1. The increased number of centrosomes in a patient with *CHAMP1* mutation.

Three color staining of centrioles in lymphoblast cells from a healthy person and a patient with *CHAMP1* mutation. The cells were stained with the indicated antibodies. The number indicates centrosomes containing mother and daughter centriole and having MTOC activity. Scale bar, 5 μm.

SUPP. REFERENCES

Piel, M., Meyer, P., Khodjakov, A., Rieder, C. L. & Bornens, M. The respective contributions of the mother and daughter centrioles to centrosome activity and behavior in vertebrate cells. *J. Cell Biol.* **149,** 317–329 (2000).
